# Quality of Life, Anxiety and Depression in Turkish Women Prior to Receiving Assisted Reproductive Techniques

**Published:** 2012-06-19

**Authors:** Gul Pinar, Hulusi Bulent Zeyneloglu

**Affiliations:** 1. Department of Nursing, Faculty of Health Science, Yıldırım Beyazit University, Ankara,Turkey; 2. Department of IVF, Faculty of Medicine , Baskent University, Ankara, Turkey

**Keywords:** Anxiety, Assisted Reproductive Techniques, Depression, Infertility

## Abstract

**Background:**

This study evaluated the quality of life and anxiety-depression levels of patients prior to receiving assisted reproductive techniques.

**Materials and Methods:**

This cross-sectional research was conducted in the *In-Vitro* Fertilization Unit of a private University’s Faculty of Medicine, Department of Obstetrics and Gynecology. Study participants consisted of 160 individuals diagnosed as infertile whose treatment plans were determined, as well as 160 reportedly healthy fertile individuals (n=320). Each participant completed the Patient Identification Form, Beck Anxiety Inventory, Beck Depression Inventory and Quality of Life Scale questionaires.

**Results:**

The results of this study indicate a higher prevalence of depression and anxiety in the infertile group (p<0.05). Also, quality of life scores were found to be lower in the infertile group (p<0.05).

**Conclusion:**

Individuals who experience infertility need psychological support in order to overcome the psycho-social difficulties they experience. It is essential to have studies that stress the importance of integrating psychological and emotional support into clinical practice.

## Introduction

Infertility is an important health problem prevalent throughout the world. There are approximately 80 million infertile couples in the world and it is suggested that this number corresponds approximately to 15% of all couples at reproductive age (18-45) in the world (1, 2). This ratio seems to be reaching even higher levels in Turkey and other developing countries. The number of infertile couples in Turkey is estimated to be about 1.5-2 million (3). Worldwide, in 55-75% cases the problem is primary infertility with the rate of secondary infertility at about 25-40% (1). Contributing to these numbers is the fact that in our modern world more and more women tend to postpone marriage and childbearing for social or career purposes. Leaving marriage to older ages has significantly increased the rate of infertility in society (4). The significant prevalence of infertility throughout the world necessitates special focus on understanding the deep and multidimensional psychological and social impacts of infertility (5-8).

Infertility is defined as the inability to conceive a child after 12 months of regular unprotected sexual intercourse (1). Although historically it has been perceived as a consequence of the female’s inability only, medical advances of our modern world have clearly demonstrated that it is a state linked not only to the female but also the male, and sometimes to both partners (7). Today, 40% of infertility is attributed to female factors while for another 40% male factors appear to be the cause of infertility (1, 2). The remaining 20% is attributed to both partners, while 10% of this 20% is not attributable to either partner. This type of infertility is called "unexplained" infertility (2).

While being mainly a medical condition, the diagnosis of infertility still has multi-dimensional effects on couples including biological, psycho-social, economical, ethical, and cultural dimensions (4-8). Particularly, studies conducted over the last 20 years have made a significant contribution to our understanding of this multi-dimensional impact structure focusing especially on the emotional aspects that couples, as well as individuals, experience (9). Although infertility is usually linked to a physical problem, the stress and loss associated with infertility can have serious impact on psychological, physical, economic, and social well-being, and thus the quality of life in general (10-12).

Drawing attention to this fact, Monga et al. referred to infertility as a developmental crisis that can threaten a couple’s future goals (9). In addition to the couples who experience this phenomenon, infertility also affects the couple’s environment, especially in societies with strong family connections (10). Studies indicate that Islamic countries demonstrate particularly rigid forms of social implications related to the problem of infertility. These societies stand out with their primary focus on childbearing as an important goal for marriage (3-5, 12-14). Thus, being unable to fulfill this primary goal an infertile marriage is highly likely to be regarded as a failure, as asserted by Ramezanzadeh et al. in their study where a comprehensive comparison of Islamic vs. non-Islamic societies’ perceptions of infertility is made.

This comparative study suggests that childbearing is a vital means of stability and satisfaction in married life in Islamic societies such as Iran while negative social attitudes to infertility may reach detrimental levels and result in the end of the marriage (divorce) or a second marriage for men. Thus, the study also reveals a strong belief in infertility as a female factor phenomenon in such societies (12). Being mainly Islamic in nature, Turkey is another country bearing similar characteristics in terms of infertility and its social implications (3-5). Insufficiency and worthlessness associated with the importance attributed to having children and the progression of the males’ family surname in such societies, are strong and widely experienced negative feelings (3, 4). Additionally, the existence of a social environment devoid of proper sensitivity to the issue around the couple aggravates the situation even further bringing the couple face to face with the society’s expectations and the social pressure resulting from these expectations. In this light, infertility may become a major crisis affecting the whole family and not only the couple (3, 4, 15, 16).

Another important point concerning these psychological and sociological impacts of infertility is the fact that although they are common among cases of infertility, certain socio-demographical and medical characteristics such as age, educational background, family type, working status, income level, and environmental pressures play important roles in determining the level of anxiety-depression experienced by the infertile group (3-5, 7, 9, 15-19).

It is also known that all the psychosocial effects of infertility become stronger as the duration of diagnosis and treatment increases (12). Therefore, it is of utmost importance that couples be psychologically supported by health care personnel at the phase of diagnosis, prior to receiving assistance and treatment (13). Especially, consultancy services given to infertile couples help individuals and couples to adapt their lifestyles and strengthen their relations. It also may help to overcome anxiety, hostility, anger, and dissatisfaction (16). However, it is also important to keep in mind that the decision of whether to start infertility treatments is also a difficult one which may provoke anxiety (20). Previous studies have reported a high prevalence of depression and anxiety disorders among women receiving infertility treatments, but the estimates vary widely (7-9, 11, 20, 21).

Taking into consideration the aforementioned facts about infertility and its psycho-social impacts as well as the importance of timely consultancy services, this study aims, on one hand, to make a comparative analysis of fertile and infertile groups in terms of anxiety-depression levels and the quality of life, and on the other, to reveal whether meaningful differences exist within the infertile group depending on the various socio-demographical characteristics they exhibit. More specifically the following two hypotheses were tested throughout this study:

1. a. Beck Depression Inventory (BDI) and Beck Anxiety Inventory (BAI) scores in the infertile group are higher than in the fertile group while quality of life scores are higher in the fertile group.
2. b. Within the infertile group certain independent socio-demographic variables determine the anxiety-depression levels as well as quality of life.

## Materials and Methods

### Format, time and place of research

This study was planned as a cross-sectional study. Research was conducted in a private University Faculty of a Medical Department of Obstetrics and Gynecology in Ankara. Required permissions were received from Baskent University Research and Ethics Committee in order to conduct the research. The patients who agreed to participate in the study and who met the research criteria were included in the study.

### Population and sampling

The population of the study consisted of 214 infertile patients who were referred to the aforementioned medical center between January 20 and May 20, 2009. Of these, 160 patients complying to the study criteria and willing to volunteer in the study were taken as the sample for the infertile group. In addition, 160 fertile people referred to the Maternity-Child Welfare Centre of the same institution, complying with the criteria, and willing to participate in the study were included in the study. Thus, a convenience sample total number of 320 people made up the complete sample of the study.

Infertility cases associated with male factors were included in the study (male=80, female=80). Study participants in the infertile group were at least elementary school graduates, above 18 years old and married, diagnosed with primary or secondary infertility (not having a living child) with planned programs of infertility treatment, receiving infertility treatment for the first time, not diagnosed with psychiatric illness before infertility diagnosis, and did not have a severe illness or receive treatment for such an illness. Those with a history of miscarriage were not excluded from study.

Volunteer fertile women and their husbands agreed to participate in the study and formed the fertile group. These women had at least one child older than one year of age, were married, did not have any difficulty conceiving without assistive reproduction techniques, were not treated for any gynecological illness, were not pregnant, and were literate.

### Data collection tools and application

Three instruments were used for data collection purposes. These were the following: "Patient Identification Form", "BAI", "BDI" and the "World Health Organization Quality of Life (WHOQOL)- Brief life Quality Scale". These were used in identifying socio-demographical characteristics and obtaining the infertility history (22-24). After treatment protocols were completed, the researchers conducted the face-to-face interview with each study participant and ensured that data collection instruments were completed. Each interview took about 30-40 minutes (both groups interviewed). Individuals in the fertile group also completed the "Healthy Individual Identification Form", "BAI", "BDI" and "WHOQOL-Brief Quality of Life Scale".

### Patient Identificatio Form

The "Patient Identification Form" was prepared by the researchers in order to determine socio-demographical characteristics (age, sex, educational background, spouse’s educational background, working status, income, family type, marriage type, smoking and alcohol use), as well as the participants’ feelings and opinions about treatment. Form items were determined from review of the literature on the experiences of individuals with infertility (4-13, 20, 21). This form consisted of 31 multiple choice and open-ended questions. Of socio-demographical characteristics, the following were questioned: duration and type of infertility, reason for infertility, treatment method, pressure of environment on infertility, having knowledge on infertility, interested to be informed on infertility and difficulties regarding with infertility treatment.

### Beck Depression Inventory

The BDI is a likert-type scale consisting of 21 items. The total scores range between 0 and 63. It is used in clinical applications in order to determine the severity of depression. Interpretation of the depression score is as follows: 0-10 points show that there is no depression, 11-17 points indicate a mild level of depression, 18-29 points indicate a medium level depression, and 30-63 points indicate severe depression Hisli conducted a validity reliability study in 1988 for Turkish application of the BDI, with Cronbach Alfa internal consistency values of 0.74 (22).

### Beck Anxiety Inventory

BAI is a likert-type clinical scale consisting of 21 articles, which was developed by Beck, Epstein, Brown, and Steer in 1988 in order to measure anxiety levels. The patient is asked to evaluate symptoms for "the last one week including today". Total scores range between 0 and 63. Ulusoy et al. determined that there is internal consistency in the use of the BAI scale with Turkish patients (Cronbach Alfa=0.81). Anxiety levels of patients were classified according to the scores received in BAI: 0-17 points indicates mild level, 18-24 points indicates medium level, and 25 and above indicates severe anxiety (23).

### World Health Organization Quality of Life

The WHOQOL has 27 questions and consists of four sub-groups, which are physical, emotional, environmental, and social areas. Questions are answered by taking into account the experience of the patient over the last 15 days. A Turkish version of the scale was translated in 1997 and is available, and can be used both for healthy individuals and patients with the method stipulated by WHO. The internal consistency of the WHOQOL scale is 0.92 as established by the WHOQOL Turkey Group (24).

### Analysis of data

Pearson correlation analysis, Student’s t test and Chi-Square were used in the evaluation of data. Means and standard deviations of BDI, BAI, and Quality of Life (QOL) scores were given in descriptive statistics. Student’s t test was applied to evaluate the relationships between socio-demographic variables and BDI, BAI, QOL scores. The same relationships were also examined through the application of the Pearson correlation analysis. Assumptions of the Multiple Regression Analysis were assessed by focusing on the BDI, BAI, QOL scores and the socio-demographic and clinical characteristics of the infertile group. P ≤ 0.05 was considered as the statistical significance level.

**Table 1:** Findings on characteristics of patients


Socio demographical	Groups				Total		Statistical analysis*
	Infertile (n=160)		Fertile (n=160)					
	n	%	n	%	n	%

**Age (Year)**							
26-29	64	40.0	72	45.0	136	42.5	x^2^=0.274 p=0.875	
30-33	40	25.0	48	30.0	88	27.5	
≥ 34	56	35.0	40	25.0	96	30.0	
**Educational background**							
Literate	32	20.0	48	30.0	80	25.0	x^2^=0.361 p=0.835	
Elementary school	44	27.5	36	22.5	80	25.0	
High school and above	84	52.5	76	47.5	160	50.0	
**Working status**							
Employment	68	42.5	84	52.5	152	47.5	x^2^=1.086 p=0.297	
Unemployment	92	57.5	76	47.5	168	52.5	
**Family type**							
Core family	112	70.0	92	57.5	204	63.7	x^2^=0.131 p=0.718	
Extended family	48	30.0	68	42.5	116	36.3	
**Smoking status**							
Yes	76	74.5	96	60.0	172	53.7	x^2^=1.111 p=0.292	
No	84	52.5	64	40.0	148	46.3	
**Alcohol use**							
Yes	60	37.5	72	45.0	132	41.2	x^2^=1.216 p=0.267	
No	100	62.5	88	55.0	188	58.8	
**Total**	160	100.0	160	100.0	320	100.0		


* Chi- Square test was used.

## Results

As can be seen from table 1, there is no significant difference between groups with respect to age (x<sup>2</sup>=0.272, p=0.873), educational background (x<sup>2</sup> =0.361, p=0.835), working status (x<sup>2</sup>=1.086, p= 0.297), family type (x<sup>2</sup>=0.131, p=0.718), smoking status (x<sup>2</sup>=1.111, p=0.292), and alcohol use habits (x<sup>2</sup>=1.216, p=0.267). The mean age of participants was 32.76 ± 3.76 (minimum: 26, maximum: 41) in the infertile group and 31.27 ± 3.76 (minimum : 24, maximum: 43) in the fertile group.

All participants were comfortable with their social status. Couples stated that they knew and understood each other before marriage and were living in an urban area. The percentage of university (or above) graduates in spouses was 66.7% in the infertile group and 63.3% in the fertile group. Considering both groups, 36.7% were married for 1-3 years, 43.3% were married for 4-6 years, and 20% were married for ≥7 years. Participants described their incomes as "medium" (46.7%) or "good" (53.3%). There was no significant statistical difference between the groups in respect to the educational background of spouses (x<sup>2</sup>=1.815, p =0.464), period of marriage (x<sup>2</sup>=1.116, p=0.553), type of marriage (x<sup>2</sup>=0.156, p=0.381), and income status (x<sup>2</sup>=0.213, p=0.305).

As can be seen from table 2, 72.5% of the infertile group participants were experiencing primary infertility, with no history of miscarriage. For 45% of them, the infertile period was "1-2 years" and the reason for the infertility for 50% was "female factor". Other findings were as follows: 40% planned " *in vitro* fertilization (IVF), hormone, and tubal operation" treatment, 65% of the infertile stated that they experienced "environmental pressure" associated with infertility, 70% had knowledge on infertility treatment, 60% needed to be informed again on the treatment plan, and 62.5% believed that they would experience difficulty with the treatment process.

**Table 2:** Findings on medical characteristics of the infertile group


Medical characteristics	N	%

**Type of infertility**		
Primary infertility	116	72.5
Secondary infertility	44	27.5
**Infertility period**		
1-2 years	72	45.0
3-4 years	48	30.0
≥ 5 Years	40	25.0
**Reason of infertility**		
Female factor	80	50.0
Male factor	80	50.0
**Infertility treatment**		
Infusion	44	27.5
Infusion and hormone	52	32.5
IVF, hormone, tubal operation	64	40.0
**Pressure of environment on infertility**		
Yes	104	65.0
No	56	35.0
**Having knowledge on infertility treatment**		
Yes	112	70.0
No	48	30.0
**Subjects which patient is interested to be informed on infertility treatment**		
Treatment plan	96	60.0
Pregnancy chance	64	40.0
**Difficulties / possibilities regarding with infertility treatment**		
Yes	100	62.5
No	60	37.5
**Total**	160	100.0


Infertile group participants were asked the question "what do you think about the meaning of having a child". Seventy-six expressed that this was "my biggest dream in my life". In addition, in response to the question "whose wish for a child is stronger, yours or your spouse’s?" 20% answered as "me", 23.3% answered as "my spouse", and 56.7% answered as "both of us". All of the individuals in the infertile group participating in the study had expected health personnel to support them during treatment process and to be interested in and receptive to their questions and concerns.

This study describes the experiences or possible difficulties with the treatment process. Accordingly, it was reported that difficulties such as failure (66.7%), psychological problems and stress (50%), fear of the operations (36.7%), necessity of frequent examinations (33.3%), and having trouble in leaving work (30%) have been or may be experienced. One hundred percent (100%) of the patients in the infertile group stated that they had the support of their spouses during the treatment process. This support was also found with "health personnel"(56.7%), family-relatives (26.7%), and friends (20%), respectively. It was determined that none of the participants in the infertile group had received support from psychiatric-mental health care providers to date.

As can be seen from table 3, the mean BAI score was 8.43 ± 4.16 and the mean BDI score was 25.00 ± 11.58 for the infertile group. Fertile group averages were 5.25 ± 3.26 (BAI) and 19.87 ± 9.78 (BDI). There was significant statistical difference between groups with respect to BAI (t=9.135, p=0.003) and BDI scores (t=11.026, p=0.006). Statistically significant differences existed between the fertile and infertile groups in terms of the psychological (t=-4.400, p=0.004), social (t=10.800, p=0.001), environmental (t=-6.112, p=0.002), and physiological (t=3.000, p=0.007) sub-dimensions of the life quality scale. Correspondingly, quality of life scores were found to be lower in the infertile-group (p<0.05).

According to table 4 there were no symptoms of depression in 35% of the infertile group while 25% showed a "mild" level of symptoms, and 40 % showed a "medium" level of depression symptoms. In the infertile group, 62.5% experienced "mild" anxiety, while 25% had "medium" and 12.5% had "severe"anxiety. Depression rates in the fertile groups were lower than those of the infertile group (x<sup>2</sup>=44.0.93, p=0.001). No significant difference was found between the two groups in terms of the levels of anxiety they experience (x<sup>2</sup>=2.459, p=0.293).

**Table 3:** Distribution of BDI, BAI, and QOL score averages of infertile and fertile groups


Test	Group		t*	P
	Infertile mean ± SD	Fertile mean ± SD		

**BAI**	8.43 ± 4.16	5.25 ± 3.26	9.135	0.003
**BDI**	25.00± 11.58	19.87 ± 9.78	11.026	0.006
**QOL-score**				
**Physical**	74.8 ± 5.78	99.2 ± 5.83	-4.400	0.004**
**Psychological**	64.70± 4.72	85.50 ± 6.85	-10.800	0.001**
**Social**	66.45± 8.17	87.57 ± 8.64	-6.112	0.002**
**Environment**	69.77± 5.25	89.93 ± 6.26	3.000	0.007**


*Student (independent sample) t test was used. ** p<0.05.

**Table 4:** BDI and BAI percentage distributions of infertile and fertile groups


Test	Group		X^2^*	P
	Infertile n (%)	Fertile n (%)		

**BDI**				
1-10- doesn’t exist	56 (35.0)	108 (67.5)		
11-17-mild	40 (25.0)	35 (22.0)	44.093	0.000**
18-29-medium	64 (40.0)	17 (10.5)		
**BAI**				
0-17-mild	100 (62.5)	112 (70.0)		
18-24-medium	40 (25.0)	29 (18.0)	2.459	0.293
≥ 25-severe	20 (12.5)	19 (12.0)		


*Student (independent sample) t test was used. ** p<0.05.

High positive correlation (r=0.308, p= 0.017) was found between depression (r=0.390, p=0.017) and anxiety scores of the infertile group while, for the same group, there was a strong negative correlation between these values and quality of life (r=-0.367, p=0.041).

The distribution of the average BDI and BAI scores of the infertile group according to certain socio-demographic characteristics (age, sex, education level, family type, and environmental pressure) is shown in table 5. Accordingly, while both genders in this group demonstrated anxiety-depression, the rates were higher among women participants. The same table shows that average BAI and BDI scores tend to decrease as the level of education increases or if the family type is core, while an opposite relationship exists between increasing ages and the same scores. The study also demonstrated higher anxiety-depression levels with subjects who were not well informed on infertility treatment, experienced longer periods of infertility, were unemployed, or got married through arranged marriage.

In table 6 the relationships between the BDI, BAI, QOL scores and independent variables were examined through a multiple regression analysis (R=0.51, R2=0.50, F=35.75, p=0.000). The analysis demonstrated that the BDI, BAI, and QOL scores in the infertile group were affected by sex (BDI: β=0.21, BAI: β=0.16, QOL: β=0.28), age (BDI: β=0.18, BAI: β=0.10, QOL: β=-0.29), education (BDI: β=-0.34, BAI: β=-0.33, QOL: β=0.11), working status (BDI: β=-0.12, BAI: β=-0.27, QOL: β=0.37), type of marriage (BDI: β=0.26, BAI: β=0.33, QOL: β=0.18), environmental pressure (BDI: β=0.19, BAI: β=0.28, QOL: β=-0.23), being informed on infertility treatment (BDI: β=-0.22, BAI: β=-0.27, QOL: β=0.16), and the duration of infertility (BDI: β=0.17, BAI: β=0.23, QOL: β=-0.12). However, education of spouse, income, sources of support, type and reason of infertility, and type of infertility treatment did not have an impact on these scores (p>0.05).

**Table 5:** Distributions of socio-demographical characteristics of infertile group with BDI and BAI score averages


Score		Min	Max	Mean ± SD

	**Sex**			
**BDI**	F	1	20	9.00± 4.72
	M	3	11	7.30± 2.58
**BAI**	F	7	44	25.60± 11.61
	M	10	41	23.80± 12.06
	Age			
**BDI**	26-29	1	18	7.85± 4.86
	30-33	4	13	7.86± 3.13
	≥ 34	6	20	9.60± 3,95
	26-29	7	44	22.92± 13.20
**BAI**	30-33	14	39	24.10± 9.57
	≥ 34	10	41	30.14± 11.00
	**Educationalbackground**			
**BDI**	Elementary school	6	11	9.57± 5.442
	High school	3	20	8.25± 2.217
	University	1	18	8.05± 4.075
	Elementary school	20	41	34.50± 9.74
	High school	7	24	26.79± 10.90
**BAI**	University	10	44	14.71± 7.15
	**Family type**			
**BDI**	Immediate family	1	20	8.42± 4.30
	Extended family	5	13	9.50± 3.6
	Immediate family	7	44	24.35± 11.47
**BAI**	Extended family	10	39	29.25± 13.15
	**Pressure of environment on infertility**			
**BDI**	Yes	1	20	8.42± 4.30
	No	5	13	9.50± 3.6
**BAI**	Yes	7	44	24.35± 11.47
	No	10	39	29.25± 13.15


**Table 6:** Multiple regression analysis of socio-demographical characteristics of infertile group with BDI-BAI and QOL score averages


Variables	Test*		
	β	t	p

**BDI**			
Sex	0.21	2.39	0.001**
Age	0.18	2.63	0.003**
Education	-0.34	-2.43	0.001**
Working status	-0.12	-3.33	0.009**
Marriage type	0.26	2.24	0.002**
Pressure of Eenvironment	0.19	2.64	0.008**
Having knowledge on infertility treatment	-0.22	-2.20	0.002**
Infertility period	0.17	3.46	0.005**
**BAI**			
Sex	0.16	2.76	.003**
Age	0.10	1.75	0.004**
Education	-0.33	-3.39	0.001**
Working status	-0.27	-2.62	0.006**
Marriage type	0.33	2.44	0.002**
Pressure of environment	0.28	2.10	0.001**
Having knowledge on infertility treatment	-0.27	-2.11	0.002**
Infertility period	0.23	2.36	0.006**
**QOL**			
Sex	0.28	3.89	0.001**
Age	-0.29	-2.35	0.003**
Education	0.11	2.30	0.001**
Working status	0.37	5.49	0.001**
Marriage type	0.18	2.71	0.002**
Pressure of environment	-0.23	-3.46	0.001**
Having knowledge on infertility treatment	0.16	2.13	0.004**
Infertility period	-0.12	-2.48	0.003**


R=0.51, R2=0.50, F=35.75, p=0.000. *Multiple regression analysis was used. **p<0.05.

## Discussion

In the study, the mean BAI and BDI scores were 8.43 ± 4.16 and 25.00 ± 11.58 for the infertile group, respectively. For the fertile group, BAI and BDI scores were 5.25 ± 3.26 and 19.87 ± 9.78, respectively. Most of the infertile subjects suffered from depression and anxiety as higher depression and anxiety scores suggest. At the same time, quality of life scores were lower in the infertile group (p<0.05).

In another study on the psychological experiences of infertile patients, conducted on a 51-person infertile and a 30-person fertile group in the Infertility Policlinics of Istanbul University Cerrahpasa School of Medicine and Marmara University School of Medicine, the average BDI was found to be 6.10 ± 6.29 in the infertile group and 7.66 ± 6.70 in the control group. In the same study the BAI average was found to be 11.6 ± 11.17 in the infertile group and 10.15 ± 9.14 in the fertile group. Thus, Gulseren et al. did not find any significant difference between infertile and fertile groups with respect to anxiety and depression (4). Fekkes et al. stressed that quality of life can be affected by infertility treatment in a positive manner (15). However, there are other studies reporting that anxiety-depression rates increased by 10-50% in infertile individuals (6, 7, 12, 16). In a study conducted in Poland, which compared infertile women to healthy pregnant women, depression rates were found to be higher in the infertile group and their quality of life was affected adversely (8).

In this study, the effects of socio-demographical and medical characteristics such as age, educational background, husband’s educational background, family type, working status, income level, environmental pressures, psychological response to infertility, and quality of life were examined. The percentage of those stating that they felt environmental pressure was high in the infertile group (65%). We found that environmental pressure increased anxiety-depression scores and decreased quality of life (p<0.05). Significant differences were found in BAI-BDI and QOL scores depending on the educational background of infertile patients. Accordingly, anxiety-depression scores of those having at least a university education were low and quality of life scores were high (p<0.05). Chachamovich et al. examined the relationship between education and quality of life and found a decrease in environmental and mental health scores in the group with lower educational status (7).

In this study, anxiety-depression scores increased in direct proportion to the increase in age in the infertile group (p< 0.05). Accordingly, it can be said that anxiety-depression is dependent on age. This finding is in agreement with the findings of other studies on the issue (12, 14-16). It should also be noted that there are studies like that of Drosdzol and Skrzypulec (8) and Monga et al. (9) that suggest a decrease in quality of life and an increase in psychological problems as age increases.

Support from partner is a crucial factor in the positive effects of infertility treatment (5). The predominately congruent perceptions of QOL within the infertile dyad may reinforce the role of couple-based interventions to reduce the negative impact of infertility (9, 14). Some researchers did not find any significant relationship between the BAI-BDI-WHO-QOL scores and type or reason of infertility, and support resources of infertility (23, 24). Some previous studies have shown that lack of spousal support was important in infertile couples with respect to their risk of anxiety-depression (10, 21). In this study, all of the patients in the infertile group stated that they had the support of their spouses.

In our study, we determined that the psychological effects of infertility become stronger as the duration of infertility increases (p< 0.05). Previous studies on this subject matter support our findings (14, 19). However, other studies show that the infertility duration does not have any effect on anxiety-depression and quality of life (p> 0.05). (8-10). Noorbala et al. used Beck’s questionnaire to study the prevalence of depression and the effect of psychiatric intervention on the rate of depression in 319 infertile couples at Vali-e-Asr Infertility Research Center. Findings showed that 48% of women and 23.8% of men suffer from various degrees of depression. Of this 48% of women, 30% suffered from mild, 12.5% from moderate, and 5.3% from severe depression and of the 23.8% of males, 16.6% suffered from mild, 4.7% from moderate and 2.5% from severe depression (20).

Coping strategies of women and men in the infertility period differ significantly according to social role theory (2). Women are more open in expressing their feelings while men tend to behave as if there is no problem. Several comparative studies conducted on the psychological situations of women and men experiencing the infertility process showed that the quality of life for men is more affected by the infertility process (6); yet, other studies showed that infertility-related anxiety level is higher and quality of life is worse in women experiencing this process (8, 14, 19). In our study both genders showed anxiety-depression while this rate is higher in women (p< 0.05). This is in compliance with Wischmann’s findings which showed that the prevalence of depression is higher among infertile women than infertile men (21).

In our study significant correlations were found between marriage type and levels of anxiety-depression (p< 0.05). Infertile women who had arranged marriages experienced anxiety more than those who were not in arranged marriages. Literature states that an increase in the period of marriage affects one’s psychological situation adversely (4, 12, 15).

We found that infertile individuals had some treatment-specific needs and difficulties. The feelings of failure, psychological problems, fear about operations, need to have frequent examinations, and troubles leaving the workplace for appointments were found to be difficulties. In another study it was found that some patients had isolation, social problems, insomnia, and stress problems (19). Albayrak and Gunay reported financial problems due to expensive fertility treatment medications, the need to leave the workplace frequently, and the need of women to adjust their work situations or career plans (5). It is very important to inform individuals about their treatment procedures. Well-informed patients are more accepting of their problems and accommodating with their treatments (3, 17). In our study, the BAI-BDI and QOL scores of those who stated that they were informed about infertility treatment were lower (p< 0.05).

## Conclusion

In this study it was determined that the infertile group had anxiety and depression related symptoms and their quality of life was affected adversely. They stated that they had faced difficulties at the beginning of their treatment process. In conclusion, our study found that individuals who are infertile need psychological support in order to overcome the difficulties they experience. This study proposes that physicians and nurses be aware about anxiety-depression disorders among infertile groups and the necessity of referring patients to psychosocial counsellors who provide professional infertility counselling. Moreover, health teams may provide a routine counselling model to understand the factors contributing to anxiety-depression and quality of life in the infertile couples. Thus, counselling may help them to cope with the negative feelings especially when their treatment duration is prolonged. Finally, comprehensive qualitative and prospective studies should be conducted in order to examine further the effects of infertility on mental health and quality of life for infertile groups.
